# Progressive amnestic cognitive impairment in a middle-aged patient with developmental language disorder: a case report

**DOI:** 10.1186/s13256-020-02483-w

**Published:** 2020-09-03

**Authors:** Masahiko Takaya, Kazunari Ishii, Kaori Kiguchi, Kazumasa Saigoh, Osamu Shirakawa

**Affiliations:** 1 Department of Neuropsychiatry, Faculty of Medicine, Kindai University, 377-2, Onohigashi, Osakasayama, Osaka, 589-8511 Japan; 2 Department of Radiology, Faculty of Medicine, Kindai University, Osaka, Japan; 3 Department of Neurology, Faculty of Medicine, Kindai University, Osaka, Japan; 4 Department of Clinical Genetics, Faculty of Medicine, Kindai University, Osaka, Japan

**Keywords:** Language disorder, Developmental disorders, Amnestic cognitive impairment, Dementia

## Abstract

**Background:**

Developmental disorder and dementia in older adults have been considered unrelated clinical entities because their timing of diagnosis differs greatly; however, recent studies have suggested an association between them. This case describes a middle-aged patient with language disorder exhibiting progressive amnestic cognitive impairment.

**Case Presentation:**

A 44-year-old Japanese man with long-term language dysfunction presented for his first-ever medical evaluation at age 36 years. Although his conversational ability had been impaired since childhood, he was able to graduate from secondary school and gain unskilled employment. At age 36 years, however, his workplace environment became more stressful, which led to behavioral problems that necessitated medical consultation. He consulted two psychiatrists in vain. At age 44 years, the third attending psychiatrist examined him in detail. The major component of his language disorder was amnestic cognitive impairment in the language domain as shown by logical memory subtests of the Wechsler Memory Scale–Revised. Magnetic resonance imaging showed normal findings for his age and no small vessel disease. Global cerebral hypoperfusion versus cerebellar blood flow was shown on (^123^I) iodoamphetamine single-photon emission computed tomography, and amyloid-β deposition was negative on positron emission tomography with ^11^C-Pittsburgh compound B. Pathologic tau accumulation was negative on ^18^F-THK5351 positron emission tomography imaging. Laboratory tests show no infections, no vitamin deficiencies, and no other diseases that may cause dementia. Clinical features, results of neurocognitive tests and neuroimaging studies showed no well-known neurodegenerative diseases. Collectively, he was diagnosed with language disorder based on the *Diagnostic and Statistical Manual of Mental Disorders*, Fifth Edition criteria. Over a 2-year follow-up period, amnestic cognitive impairment in visual and language domains progressed in parallel with global cerebral hypoperfusion.

**Conclusion:**

This case suggests a possible link between language disorder as defined by *Diagnostic and Statistical Manual of Mental Disorders*, Fifth Edition criteria and progressive amnestic cognitive impairment in middle age, which may ultimately lead to dementia, derived from a neurodegenerative disease.

## Background

Developmental disorder is considered to be a congenital disease and can usually be diagnosed in childhood, whereas dementia is typically diagnosed in previously functioning older adults, suggesting that these clinical entities are unrelated. However, recent studies have suggested that developmental disorders increase the risk of later dementia [1, 2].

Here, a case of progressive amnestic impairment is described in a middle-aged man with long-term language disorder. This case report aims to investigate the possible relationship between developmental language disorder and dementia.

## Case presentation

A 36-year-old Japanese man was referred for medical consultation because of behavioral problems at work, suspected to be caused by his communication difficulties. His history was negative for injury or illness from birth through childhood, and he had no previous diagnoses of any psychiatric disorders, including developmental disorder. His relevant family history was unknown. He successfully graduated secondary school, with no obvious impairments in literacy, and was working as a school janitor at the time of referral. No personality disorders were observed. However, his communication skills were weak, which was considered to be the main cause of his social difficulties at work. However, his life-long language disorder was primarily masked because he was seldom in social situations. He reported occasional alcohol intake and tobacco smoking, but neither to excess.

His workplace stress had recently increased, which was suspected to have likewise increased his communication difficulties. Specifically, he was increasingly unable to follow instructions. After an initial examination by a neurologist revealed no organic causes for his symptoms, he was referred to a psychiatrist at the authors’ clinic. On intake assessment, it was noted that he muttered to himself and communicated poorly with others. Although aphasia was suspected, no diagnosis was given at that time.

At follow-up 2 years later, when our patient was 38 years of age, his Mini-Mental State Examination (MMSE) score was 28/30, which was within normal limits, and his cognitive subscale score of the Alzheimer’s Disease Assessment Scale–Japanese version (ADAS-cog) was 11.6/70, which was borderline between cognitive normal and mild cognitive impairment [3, 4]. A second psychiatrist took over his case. This psychiatrist conducted examinations regularly for the next 3.5 years before reducing his visits to once per year. He continued this annual schedule from age 41 to 44 years. Again, no diagnosis was given at that time.

At age 44 years, our patient again encountered workplace difficulties under a new boss. Our patient’s wife reported that, according to his boss, he could no longer manage working and the boss found him smoking in a nonsmoking area on one occasion. A third psychiatrist was consulted and asked our patient to explain the episode, which he answered with a superficial statement and noted that he considered it to be “somebody else’s problem.” The attending psychiatrist at the consultation also noted that our patient had a limited vocabulary, spoke in incomplete sentences, and often provided responses that were difficult to understand; our patient also seemed to have few social activities. Further questioning confirmed that these communication and social problems had been present since childhood, consistent with his slightly poor school record and limited occupational abilities. According to his wife, this disorder was present when they first met over 20 years earlier. The attending psychiatrist suspected the diagnosis of language disorder based on the *Diagnostic and Statistical Manual of Mental Disorders* (DSM), Fifth Edition (DSM-V) criteria, and that the developmental disorder had been masked since infancy because our patient was not active in settings that required social communication.

The psychiatrist developed a strategy to determine whether these symptoms were psychogenic or organic in origin. Laboratory results showed no remarkable findings and were negative for syphilis, human immunodeficiency virus, and human T-cell lymphotropic virus type 1 and type 2. Concentrations of thyroid-stimulating hormone, free triiodothyronine, free thyroxine, antinuclear antibody, vitamin B1, vitamin B2, vitamin B12, and folic acid were all normal. Otorhinolaryngology testing confirmed normal hearing, and neurologic testing found no evidence of Parkinsonism. In addition, neither dysarthria nor dysphemia were observed.

Therefore, additional neurocognitive, psychological, and neuroimaging tests were performed to identify neurodevelopmental/communication disorders, including the following: MMSE, ADAS-cog, Frontal Assessment Battery (FAB), Wechsler Adult Intelligence Scale–Third Edition (WAIS-III), Wechsler Memory Scale–Revised (WMS-R), Japanese Standard Language Test of Aphasia and Japanese Raven’s Coloured Progressive Matrices (RCPM) [5, 6], Parent-Interview ASD Rating Scale–Text Revision (PARS-TR) obtained with the help of his wife, and Autism-Spectrum Quotient (AQ). Total scores were as follows: MMSE, 27/30, indicating the upper limit of mild cognitive impairment; ADAS-cog, 17.3/70, suggesting Alzheimer disease (AD); and FAB, 13/18, which is within normal limits [4, 7, 8]. Subset scores were as follows: MMSE delayed recall score, 2/3, suggesting slight impairment of recent memory; ADAS-cog subtests, 8/10 for word recall, 3/5 for both expressive language and language comprehension, and 2/5 for commands [7]; and FAB verbal fluency, 0/3. Collectively, these results indicated difficulty in language usage and memory. Although the ADAS-cog results suggested the possibility of AD, there was no evidence of dementia according to the MMSE.

Table 1 shows results of the other neurocognitive tests. At baseline age of 44 years, there was a discrepancy between verbal and visual memory indices on the WMS-R. Results of logical memory I and II subscales were at the third and first percentiles for his age, respectively, and were much lower than those for visual reproduction I and II. No significant discrepancies were observed among group indices of the WAIS-III at baseline, and all the group indices were under 100. Alternatively, his RCPM score for nonverbal neurocognitive performance was much better than average for his age [6]. His AQ was 31, but he did not meet DSM-V criteria for autism-spectrum disorder (ASD) [9]; furthermore, the PARS-TR results also provided no evidence of ASD. In addition, the Tokyo version of the Childhood Autism Rating Scale was administered [10]; however, reliable data could not be obtained because of poor communication between patient and examiner. Other baseline tests and imaging studies were performed to exclude specific organic disorders and are discussed in the context of the 2-year follow-up results for comparison.

**Table 1:** The results of neurocognitive tests

	Baseline	After 2 years
WAIS-III		
FSIQ	76 (5 percentile)	77 (6 percentile)
VIQ	72 (3 percentile)	71 (3 percentile)
PIQ	84 (14 percentile)	88 (21 percentile)
VCI	78 (7 percentile)	78 (7 percentile)
POI	87 (19 percentile)	95 (37 percentile)
WMI	85 (16 percentile)	81 (10 percentile)
PSI	81 (10 percentile)	89 (23 percentile)
WMS-R		
VerM (Index)	68	54
VisM (Index)	96	96
GM (Index)	73	62
AtCo (Index)	110	94
DM (Index)	70	< 50
DS forward	7 (54 percentile)	8 (68 percentile)
DS backward	6 (56 percentile)	6 (56 percentile)
VS forward	11 (91 percentile)	9 (66 percentile)
VS backward	10 (85 percentile)	6 (23 percentile)
LM I	7 (3 percentile)	2 (1 percentile)
LM II	1 (1 percentile)	0 (1 percentile)
VR I	31 (14 percentile)	34 (23 percentile)
VR II	25 (16 percentile)	7 (1 percentile)
RCPM		
(out of 36)	36	34

*AtCo* Attention/Concentration, *DM* Delayed Memory, *DS* Digit Span, *FSIQ* Full-Scale IQ, *GM* General Memory, *LM* Logical Memory, *PIQ* Performance IQ, *POI* Perceptual Organization Index, *PSI* Processing Speed Index, *RCPM* Raven’s Coloured Progressive Matrices, *VCI* Verbal Comprehension Index, *VerM* Verbal Memory, *VisM* Visual Memory, *VIQ* Verbal IQ, *WMI* Working Memory Index, *VR* Visual Reproduction, *VS* Visual Span, *WAIS-III* Wechsler Adult Intelligence Scale–Third Edition, *WMS-R* Wechsler Memory Scale–Revised

At follow-up 2 years later, when our patient was 46 years of age, delayed recall index and visual reproduction II percentiles of the WMS-R had decreased from baseline, and delayed recall impairment was apparent in both verbal and visual domains (Table 1). The results of other neurocognitive tests were not remarkably different from those at baseline (Table 1).

**Fig. 1:**
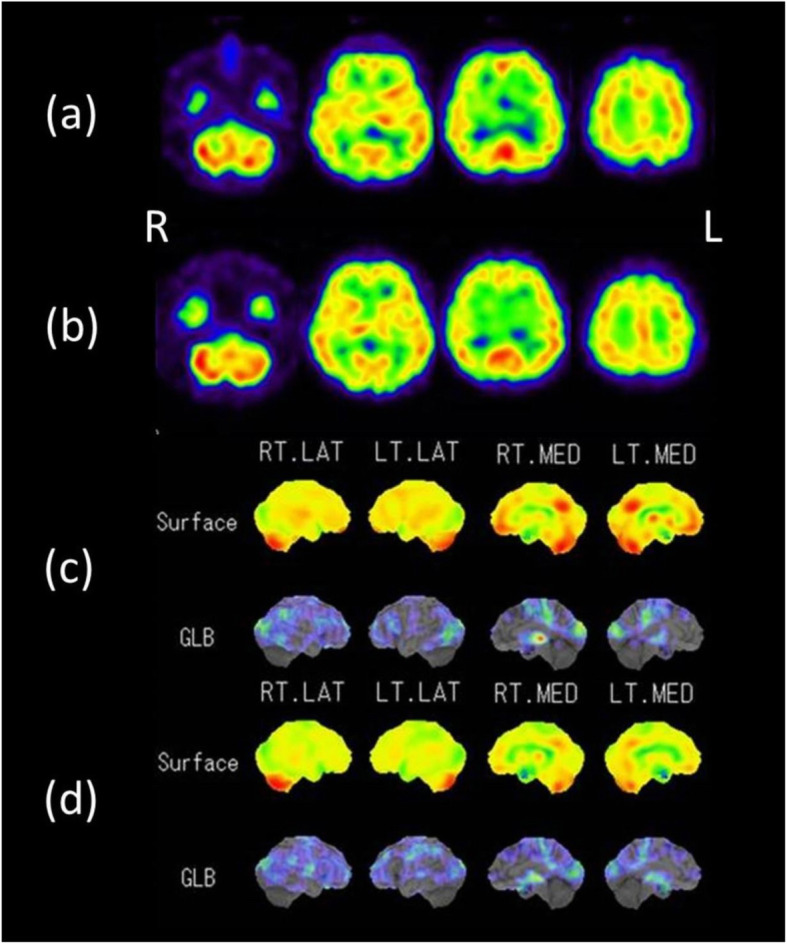
(^123^I) Iodoamphetamine single-photon emission computed tomography images showing the progression of cerebral hypoperfusion. **a** Axial images at baseline (age 44 years); (**b**) axial images 2 years later; (**c**, **d**) corresponding voxel-based analysis (using three-dimensional stereotactic surface projection software) of images (**a**) and (**b**), respectively

To exclude specific organic disorders, such as AD, dementia with Lewy bodies (DLB), frontotemporal dementia (FTD), and other types of dementia, most of the following neuroimaging tests were performed when our patient was age 44 years (baseline) and again at 46 years: magnetic resonance (MR) imaging, (^123^I) iodoamphetamine single-photon emission computed tomography (^123^I-IMP-SPECT), ^18^F-2-fluoro-2-deoxy-D-glucose (FDG)-positron emission tomography (PET), ^11^C-Pittsburgh compound B (PiB)-PET amyloid imaging, ^18^F-THK5351 PET tau imaging, and ^123^I-metaiodobenzylguanidine (MIBG) myocardial scintigraphy. No abnormalities were observed on MR images at either baseline assessment or 2-year follow-up; the images were almost completely normal and did not show small vessel disease. Only age-related slight atrophies were observed at either baseline assessment or 2-year follow-up. Similarly, FDG-PET imaging at baseline showed no evidence of neurodegenerative disease; however, baseline glucose uptake in the cerebrum was reduced compared to that in the cerebellum. Although FDG-PET was not conducted again after 2 years, ^123^I-IMP-SPECT revealed global hypoperfusion at both baseline and 2 years later (Fig. 1). Moreover, posterior cingulate perfusion had decreased further at 2 years after baseline as demonstrated by statistical voxel-based analysis using three-dimensional stereotactic surface projection software. No pathologic amyloid-β deposition on PiB-PET imaging was shown at either time [11]. Slight tau accumulation was noted in the bilateral medial temporal lobes on ^18^F-THK5351 PET imaging at both times, but this finding was not considered pathologic. Myocardial scintigraphy, which was performed only at baseline, showed normal MIBG uptake at both early and delayed images (heart/mediastinum ratios of 2.72 and 3.09, respectively).

No genetic testing for young-onset dementia was performed because the results of PiB-PET imaging and ^18^F-THK5351 PET imaging excluded AD, and because several other considerable types of dementia derived from neurodegenerative diseases, including DLB, Parkinson disease (PD), FTD, idiopathic normal pressure hydrocephalus (iNPH), corticobasal degeneration (CBD), and progressive supranuclear palsy (PSP), were also excluded based on clinical features and the results of MR images, ^123^I-IMP-SPECT, and MIBG scintigraphy.

Collectively, these investigations suggest that progressive global cerebral dysfunction underlies our patient’s communication disorder. This finding is also supported by our patient’s wife who had noted his communication difficulties since they first met 20 years earlier. On the basis of this history and recent test results, language disorder according to DSM-V and progression of both visual and verbal memory impairments were diagnosed (Table 1) [9].

## Discussion and conclusions

This case suggests the possibility of an association between language disorder and dementia based on longitudinal observational studies.

The term *developmental disorder* first appeared in the DSM, Third Edition, Revised (DSM-III-R). Subsequently, *developmental disorder* was removed as a diagnostic category in the DSM, Fourth Edition (DSM-IV) but reappeared in the DSM-V as *neurodevelopmental disorders*, a category including intellectual disabilities, communication disorders, ASD, attention deficit hyperactivity disorder (ADHD), specific learning disorder, motor disorders, and other neurodevelopmental disorders [9]. Alternatively, the term *pervasive developmental disorder* was removed in the DSM-V. For simplicity, this current report uses the term *language disorder*, which is a subitem of *neurodevelopmental disorders* according to DSM-V [9]. However, the term *developmental language disorder* has been adopted in its title for clarifying a patient’s clinical features.

Previous research has suggested relationships between specific developmental disorders and forms of dementia, such as Asperger syndrome and frontotemporal lobar degeneration [1] or PD and ADHD [2]. Here, the case of a patient with a long-term communication disorder who developed progressive amnestic cognitive impairment during middle age, suggesting a relationship between language disorders and dementia, is reported. Further research is warranted to examine if language and other developmental disorders increase the risk for later dementia. This current patient could progress to one type of dementia, which is derived from a neurodegenerative disease. Although he experienced progressive amnestic cognitive impairment, Down syndrome, for which the pathology is considered to be similar to AD, was not suspected at all because his clinical features were not compatible with those of Down syndrome.

Dementias derived from neurodegenerative diseases are classified into three types: first, those in which remarkable cerebral atrophy can be observed; second, those in which no remarkable regional cerebral atrophies can be observed; and, third, those in which the brain anatomy is severely distorted. For example, the first types of diseases are AD, FTD (Pick type), CBD, and PSP; the second types are DLB, PD, and FTD (non-Pick type); and the third type is iNPH. It is well known that regional cerebral hypoperfusion can be observed in all three types of diseases. Considering this context, this current case could be classified into the second type described but, to date, cannot be definitively diagnosed as DLB, PD, or FTD (non-Pick type). In this sense, it is possible that this patient may progress to a little-known type of neurodegenerative disease. However, the concrete mechanism for this progression cannot yet be described. The Landscape Montage Technique, which is one of the well-known art therapy techniques, may help evaluate the current patient’s clinical implications and diagnose him at an earlier stage [12].

The limitation of this current report is that it describes only one case. Therefore, many more cases presenting with language disorder must be longitudinally investigated for a better future understanding of this potential association.

## Data Availability

Data sharing is not applicable to this article because no datasets were generated or analyzed during the current study.

## Abbreviations

AD: Alzheimer disease
ADAS-cog: The cognitive subscale of Alzheimer’s Disease Assessment Scale–Japanese version
ADHD: Attention deficit hyperactivity disorder
AQ: Autism-Spectrum Quotient
ASD: Autism-spectrum disorder
CBD: Corticobasal degeneration
DLB: Dementia with Lewy bodies
DSM: *Diagnostic and Statistical Manual of Mental Disorders*
DSM-V: *Diagnostic and Statistical Manual of Mental Disorders*, Fifth Edition
FAB: Frontal Assessment Battery
FDG: ^18^F-2-fluoro-2-deoxy-D-glucose
FTD: Frontotemporal dementia
^123^I-IMP-SPECT: (^123^I) Iodoamphetamine single-photon emission computed tomography
iNPH: Idiopathic normal pressure hydrocephalus
MIBG: ^123^I-metaiodobenzylguanidine
MMSE: Mini-Mental State Examination
MR: Magnetic resonance
PARS-TR: Parent-Interview ASD Rating Scale–Text Revision
PD: Parkinson disease
PiB: ^11^C-Pittsburgh compound B
PET: Positron emission tomography
PSP: Progressive supranuclear palsy
RCPM: Japanese Raven’s Coloured Progressive Matrices
WAIS-III: Wechsler Adult Intelligence Scale–Third Edition
WMS-R: Wechsler Memory Scale–Revised
